# Correction: Symptom Experienced Three Years after Liver Transplantation under Immunosuppression in Adults

**DOI:** 10.1371/annotation/161a6145-d670-408a-a9fc-a1107b57724a

**Published:** 2013-12-10

**Authors:** Chaoying Wang, Genshu Wang, Huimin Yi, Jianling Tan, Chi Xu, Xiaocui Fang, Yang Yang, Hua Li, Qier Chen, Guihua Chen

The affiliations of the ninth and last authors are incorrect. Qier Chen is not affiliated with institution number 2, but rather institution number 3, School of Nursing, Sun Yat-sen University, Guangzhou, China. Guihua Chen is not affiliated with institution number 1, but rather institution number 2, Department of Hepatic Surgery, The Third Affiliated Hospital of Sun Yat-Sen University, Guangzhou, China. 

